# Risk of cognitive disorders and cardio-cerebrovascular diseases after electroconvulsive therapy in patients with affective and psychotic disorders

**DOI:** 10.1192/j.eurpsy.2025.556

**Published:** 2025-08-26

**Authors:** H.-C. Kim

**Affiliations:** Psychiatry and Brain Research Institute, Keimyung University School of Medicine, Daegu, Korea, Republic Of

## Abstract

**Introduction:**

Although electroconvulsive therapy (ECT) is one of the most important treatments for major mental disorders, many patients are concerned about the risk of developing cognitive impairment or cardio-cerebrovascular disease after ECT.

**Objectives:**

This study aimed to compare the incidence of cognitive disorders and cardio-cerebrovascular diseases in the ECT group and the control group at a university hospital.

**Methods:**

The subjects of this study were the ECT group (n = 173 people) who received ECT in patients with major affective or psychotic disorders, and the control group (n = 11,444 people) who did not receive ECT. The ECT and control groups were matched for demographic and clinical characteristics in a 1:5 ratio. This study investigated the incidence of cognitive disorders and cardio-cerebrovascular diseases through retrospective follow-up for up to 5 years after ECT. Statistical analysis used a multivariate Cox proportional hazards model to obtain the hazard ratio (HR) and 95% confidence interval (CI).

**Results:**

The incidence rates per 1,000 patient-years in the ECT vs. control groups were 17.56 vs. 6.25 for cognitive disorders, 4.41 vs. 4.35 for cardiovascular diseases, and 2.28 vs. 2.48 for cerebrovascular diseases. The ECT group tended to have a higher incidence of cognitive disorders compared to the control group, but this was not statistically significant (HR, 2.46; 95% CI, 0.89–6.36; p=0.07). There was no significant difference in the risk of cardiovascular disease (HR, 1.50; 95% CI, 0.21–7.09; p=0.65) or cerebrovascular disease (HR, 0.96; 95% CI, 0.05–6.56; p=0.97) between the two groups.

**Conclusions:**

This study showed that there were no significant differences in the incidence of cognitive disorders and cardio-cerebrovascular diseases between patients with major affective or psychotic disorders who received ECT and those who did not. This study suggests that ECT is a safe treatment, but further prospective multicenter collaborative follow-up studies are required to confirm this.

**Disclosure of Interest:**

None Declared

